# Identification of Shared Genetic Variants and Haplotypes Associated With Schizophrenia and Depression

**DOI:** 10.1002/brb3.71554

**Published:** 2026-06-14

**Authors:** Morteza Gholami

**Affiliations:** ^1^ Department of Paramedicine, Amol School of Paramedicine Mazandaran University of Medical Sciences Sari Iran; ^2^ Endocrinology and Metabolism Research Center, Endocrinology and Metabolism Clinical Sciences Institute Tehran University of Medical Sciences Tehran Iran

**Keywords:** depression, GWAS, haplotype, schizophrenia, variant

## Abstract

**Introduction:**

Depression is highly prevalent among individuals with schizophrenia and significantly influences disease progression, treatment response, and risk of suicide. Despite its clinical relevance, the genetic underpinnings of depression comorbidity in schizophrenia remain poorly understood. This study aimed to identify shared genetic variants and haplotypic structures contributing to the susceptibility of both conditions.

**Methods:**

We systematically screened the GWAS catalog to identify genome‐wide significant variants shared between schizophrenia and depressive disorders. Linkage disequilibrium (LD) data from the 1000 Genomes Project were subsequently used to identify overlapping LD‐associated variants, followed by haplotype reconstruction using Phase 3 genotyping data. Finally, functional analyses including brain expression quantitative trait loci (eQTL) mapping and protein–protein interaction (PPI) network analysis of genes associated with the identified variants were performed to explore their potential biological relevance.

**Results:**

Twelve shared GWAS variants were identified (*p* < 5×10^−^^6^), among which two haplotype blocks were associated with an increased risk of both disorders, including the GTCG haplotype containing rs589249 and the GG haplotype containing rs10767735. These were integrated into a novel composite structure (GTCG–GG), which may serve as a predictive marker for the co‐occurrence of schizophrenia and depression. Additionally, rs13218591 (on BTN3A1) and rs75782365 (on BTN3A2) emerged as key shared variants (SNP *p* < 5×10^−^^8^, PPI = 0.99).

**Conclusion:**

These findings highlight potential genetic markers that may contribute to the overlapping etiology of schizophrenia and depression. The identified haplotypic structures offer promising candidates for future diagnostic panels and provide a basis for further investigating of the genetic mechanisms underlying comorbidity in psychiatric disorders.

## Graphical abstract

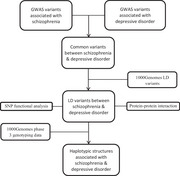

## 1 Introduction

Schizophrenia is a chronic and severe psychiatric disorder characterized by profound disturbances in thought processes, perception, and emotional responsiveness. It affects approximately 1 in 222 adults globally and disproportionately impacts individuals from lower socioeconomic backgrounds (Janoutová et al. 2016; World Health Organization 2025). Epidemiological data indicate that men tend to experience an earlier onset of schizophrenia and have a higher lifetime risk compared to women (American Psychiatric Association 2023; Janoutová et al. 2016). This disorder not only significantly impairs functional capacity but also reduces life expectancy by 10 to 25 years, largely due to comorbid medical conditions and increased risk of premature mortality (Vohra 2020).

Depression, another prevalent mental health condition, substantially affects quality of life and contributes significantly to the global disease burden. It is estimated that approximately 5% of the adult population suffers from depression, with a higher prevalence among women than men, and is recognized as a leading cause of suicide worldwide (Cuijpers et al. 2020; World Health Organization 2023). Notably, around 40% of individuals diagnosed with schizophrenia also experience depressive symptoms, highlighting a significant overlap between these two disorders (Upthegrove et al. 2017). The presence of depression in patients with schizophrenia is associated with poorer clinical outcomes, including an increased risk of suicide and challenges in the management and long‐term treatment of schizophrenia (Conley et al. 2007; Dutta et al. 2011).

While previous large‐scale studies have established a shared genetic basis between schizophrenia and major depressive disorder, most investigations have primarily focused on individual loci or polygenic risk scores (Owen et al. 2025; Tao et al. 2024; Xie et al. 2025). A more detailed characterization of the shared genetic architecture underlying these disorders remains needed to better understand the extent and biological relevance of their genetic overlap, particularly given their frequent co‐occurrence and the clinical implications for disease progression and treatment response. In the present study, we systematically investigate the genetic overlap between schizophrenia and depression by integrating genome‐wide association study (GWAS) data with linkage disequilibrium (LD) information, haplotype structure analysis, and functional annotation approaches. By extending beyond single‐variant associations, this framework aims to identify shared genomic regions and functionally relevant genetic factors that may contribute to the overlapping biology of these disorders and may support future investigations into shared pathogenic mechanisms.

## 2 Methods

### 2.1 Study Pipeline

**FIGURE 1:**
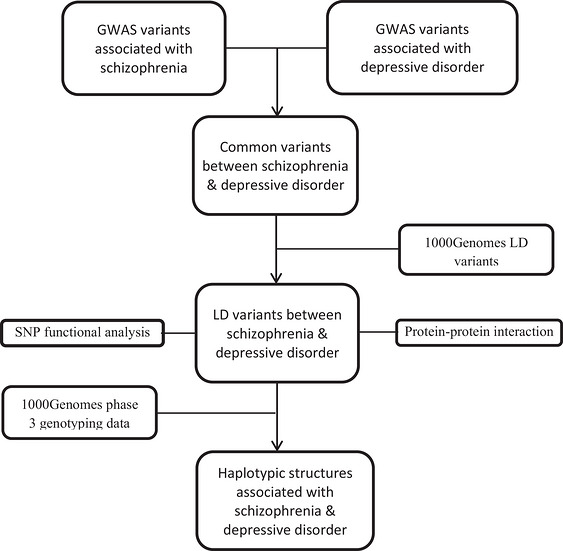
Study pipeline.

The genome‐wide significant (*p* < 5 × 10^−^^8^) and suggestive significant (*p* < 5 × 10^−^^6^) variants related to schizophrenia or depressive disorders (Wang et al. 2022) were retrieved from the GWAS Catalog. Subsequently, common variants shared between schizophrenia and depressive disorder were identified. LD variants from the 1000 Genomes Project were utilized to detect shared LD variants between the two disorders. Haplotypic structures associated with schizophrenia and depressive disorder were then characterized using genotyping data from 1000 Genomes Project Phase 3. Finally, functional annotation and protein–protein interaction (PPI) analyses were performed. The overall study pipeline is illustrated in Figure 1.

### 2.2 Common LD Variants Between Schizophrenia and Depressive Disorder

The GWAS Catalog from EMBL‐EBI (gwas_catalog_v1.0.2‐associations_e109_r2023‐05‐07) was downloaded from https://www.ebi.ac.uk/gwas/ to identify GWAS variants associated with schizophrenia or depressive disorders (Cerezo et al. 2025). The “disease/treatment” and “mapped variants” sections were used to extract variants related to these disorders. The R programming language (version 4.2.1) was used to identify common variants associated with schizophrenia or depressive disorders, hereafter referred to as index variants. HaploReg v4.2 (https://pubs.broadinstitute.org/mammals/haploreg/haploreg.php) was used to identify proxy variants in perfect linkage disequilibrium (*r* ^2^ = 1 and *D*’ = 1) with the index variants (Ward and Kellis 2016). The index and proxy variants were then combined to form the set of common GWAS LD variants associated with schizophrenia or depressive disorder.

### 2.3 Haplotypic Structures Associated With Schizophrenia and Depressive Disorder

The Phase 3 genotyping data from the 1000 Genomes Project related to common GWAS LD variants associated with schizophrenia or depressive disorder were downloaded from the Ensembl Genome Browser release 112 (https://asia.ensembl.org/index.html) (Dyer et al. 2025). Haplotypic blocks and LD plots were generated using HaploView version 4.2 (Barrett et al. 2005).

### 2.4 Functional Analysis

Variant genomic positions, variant types, and corresponding gene annotations were retrieved from the dbSNP database (https://www.ncbi.nlm.nih.gov/snp/). To assess the potential regulatory roles and biological impacts of the identified variants, comprehensive functional annotation was performed using RegulomeDB (https://regulomedb.org/regulome‐search/). Variants were prioritized and ranked based on their predicted regulatory function scores (Dong et al. 2023). Subsequently, expression quantitative trait loci (eQTL) analysis was conducted using data from the Genotype‐Tissue Expression (GTEx) project (https://gtexportal.org/home/, accessed July 14, 2025) to evaluate whether the identified variants influence gene expression levels in relevant brain tissues (Consortium 2020). This step enabled the identification of variants with significant regulatory effects on gene expression, providing insight into their potential functional mechanisms in the central nervous system. Furthermore, PPI network analysis was performed specifically on genes linked to shared GWAS variants between schizophrenia and depression to investigate whether the proteins encoded by these genes interact with each other. STRING database version 12.0 was used to construct the PPI networks and assess potential molecular interactions underlying the comorbidity of the two disorders. Notably, a stringent confidence score cutoff of 0.9 was applied for the PPI network analysis to include only highly reliable protein interactions (Szklarczyk et al. 2023).

## 3 Results

Based on GWAS data, a total of 900 genetic variants were identified as being associated with depressive disorders, and 2721 variants were associated with schizophrenia (Supplementary Results 1). Among these, 12 GWAS variants were found to be common to both schizophrenia and depressive disorders, as shown in Table 1. Additionally, three LD blocks were identified using data from the 1000 Genomes Project (Supplementary Results 2). Half of these shared variants were intronic eQTLs with strong RegulomeDB rankings, indicating potential regulatory relevance. PPI analysis revealed a robust association between proteins BTN3A2 and BTN3A1, with an interaction score of 0.99. Notably, these genes contain significant variants, rs13218591 and rs75782365, which are associated with both schizophrenia and depressive disorders in the European population (*p*‐value < 2×10^−^^8^). Further haplotype analysis based on 1000 Genomes genotyping data identified two haplotypes anchored by GWAS index variants.

**TABLE 1:** Common GWAS variants between schizophrenia and depression.

Variants	Gene	Position	Type of variant	eQTL	RegulomeDB ranking	*p* value schizophrenia	Depression
rs848293	VRK2	2:58155355	intron_variant	—	1b	Asian/9×10^−17^	EUR/9×10^−8^
rs6019826	KCNB1, LOC105372649	20:49426433	downstream_variant, intron_variant	—	1f	EUR/2×10^−6^	EUR/9×10^−9^
rs13218591	BTN3A2	6:26376604	3_prime_UTR_variant	Yes	1f	2×10^−12^	EUR/2×10^−8^
rs589249	LOC105378648, LOC107984941	1:36696751	intron_variant, upstream_variant	Yes	5	EUR/3×10^−7^	EUR,Asian/5×10^−9^
rs10767735	LINC02758	11:28621014	intron_variant	—	5	EUR, AFR, Latin/5×10^−11^	EUR/7×10^−7^
rs75782365	BTN3A1	6:26408323	intron_variant	Yes	1f	EUR/2×10^−27^	EUR/6×10^−9^
rs55834529	—	6:27104763	—	Yes	6	EUR/9×10^−27^	EUR/3×10^−9^
rs12129573	—	1:73302683	—	—	7	EUR,Asian/2×10^−12^	EUR/2×10^−12^
rs5758265	L3MBTL2	22:41221893	intron_variant	Yes	1d	8×10^−10^	EUR/8×10^−9^
rs61902811	LOC105369501	11:113500036	intron_variant	—	1f	EUR/8×10^−17^	EUR/4×10^−39^
rs4702	FES, FURIN	15:90883330	upstream_variant	Yes	1f	EUR/6×10^−25^	EUR/2×10^−12^
rs11682175	—	2:57760458	—	—	3a	EUR/1×10^−12^	EUR/5×10^−9^

**FIGURE 2:**
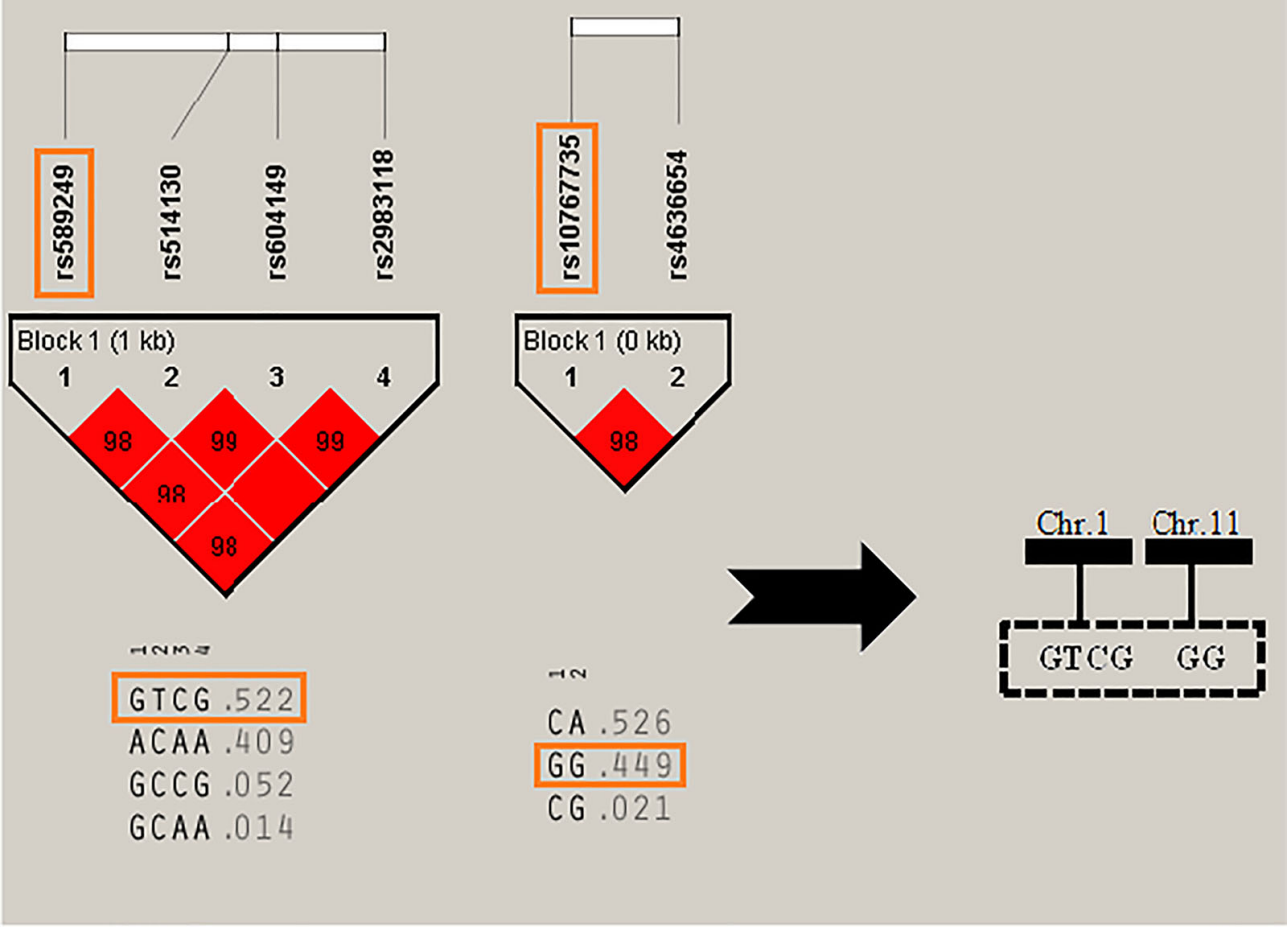
LD plots and haplotypic structures common between schizophrenia and depressive disorder.

Two haplotypic structures and corresponding LD blocks associated with an increased risk for the co‐occurrence of depressive disorders and schizophrenia were identified. The first haplotypic structure, GTCG (rs589249‐G, rs514130‐T, rs604149‐C, rs2983118‐G), is linked to the GWAS variant rs589249 located within the LOC105378648 and LOC107984941 genes on chromosome 1. The second haplotypic structure, GG (rs10767735‐G, rs4636654‐G), is associated with the GWAS variant rs10767735 within the LINC02758 gene on chromosome 11. The frequencies of the GTCG and GG haplotypes differed among the general, European, Asian, and American populations. The frequency of the GTCG haplotype was 0.522, 0.320, 0.584, and 0.488, respectively, while the frequency of the GG haplotype was 0.449, 0.603, 0.197, and 0.643, respectively. None of these haplotypes were observed in African population. The haplotypes and their frequencies in different populations are presented in Supplementary Results 3. These two haplotypic structures on chromosomes 1 and 11 represent a novel genetic framework for predicting the co‐occurrence of schizophrenia and depressive disorders. The detailed results are presented in Figure 2.

## 4 Discussion

The critical role of depression in influencing the clinical course, treatment response, and risk of suicide in individuals with schizophrenia is well established. However, to date, no definitive genetic markers have been identified that can predict susceptibility to depressive symptoms specifically in the context of schizophrenia. This knowledge gap presents a significant challenge for the development of targeted therapeutic strategies and personalized treatment approaches. Therefore, the present study represents a novel effort to investigate the shared genetic architecture underlying the co‐occurrence of schizophrenia and depressive disorders. By integrating GWAS data, LD analysis, and haplotype structure modeling, this study aims to identify potential genetic markers that may contribute to this comorbidity, ultimately informing both early detection and precision medicine strategies.

Using genotyping data from the 1000 Genomes Project, two haplotypes based on GWAS index variants were identified as potentially contributing to the co‐occurrence of depressive disorders and schizophrenia. Among these, the haplotype GTCG comprises four key variants, rs589249, rs514130, rs604149, and rs2983118, located within regions encoding noncoding RNAs. Prior GWAS studies have established a significant association between rs589249 and both schizophrenia (Aberg et al. 2013) and depression (Giannakopoulou et al. 2021) underscoring its potential relevance in the shared genetic basis of these disorders. In contrast, the roles of the proxy variants rs514130, rs604149, and rs2983118, as well as their related noncoding genes *LOC105378648* and *LOC107984941*, remain largely uncharacterized in the context of psychiatric conditions. Given the growing recognition of noncoding RNAs in the regulation of gene expression and neural function, these findings highlight novel candidate loci that may influence susceptibility to schizophrenia and depressive disorders, as well as their pathophysiology. Further functional studies are warranted to elucidate the biological mechanisms through which these noncoding variants contribute to disease risk and to assess their potential as biomarkers for comorbid presentations.

The haplotypic GG structure is characterized by the presence of two key variants, designated here as variant rs10767735 and variant rs4636654, both located within noncoding RNA regions. Previous GWAS studies have implicated variant rs10767735 in the pathogenesis of both schizophrenia (Trubetskoy et al. 2022) and depression (Howard et al. 2018), suggesting its potential involvement in the shared genetic architecture of these psychiatric disorders. In contrast, the functional significance of the proxy variant rs4636654, as well as its associated long noncoding RNA gene, *LINC02758*, remains unexplored in the context of schizophrenia and depressive disorders. Considering the emerging role of long noncoding RNAs in gene regulation and neuropsychiatric disease mechanisms, these findings point to *LINC02758* as a novel candidate for further investigation. Elucidating the contribution of variant rs4636654 and *LINC02758* could enhance our understanding of the molecular basis underlying the comorbidity of schizophrenia and depression and may provide new avenues for targeted therapeutic interventions.

Additionally, a strong PPI was observed between BTN3A2 and BTN3A1, whose encoding genes contain significant variants, namely rs13218591 (Nagel et al. 2018; Wu et al. 2020) and rs75782365 (Howard et al. 2018; Wang et al. 2022), both of which have been associated with schizophrenia and depression in populations of European descent. Previous studies have suggested that elevated expression of BTN3A2 may contribute to schizophrenia risk by modulating excitatory synaptic function (Wu et al. 2019). More recent research has also linked BTN3A2 to depressive disorders (Kang et al. 2025), highlighting its potential role in the shared pathophysiology of these conditions. Furthermore, the role of BTN3A1 as a genetic locus implicated in the comorbidity of schizophrenia and depression has been supported by findings from a recent Mendelian randomization study (Tao et al. 2024). Collectively, these data underscore the importance of BTN3A1 and BTN3A2 as promising candidates for understanding the molecular mechanisms underlying the genetic overlap and comorbidity of schizophrenia and depressive disorders.

The identification of common variants and haplotype structures shared between schizophrenia and depression has diverse potential clinical applications and paves the way for future studies to integrate genetic information into clinical practice for these diseases. These markers are potentially useful in several ways, including identifying individuals at higher risk of developing these diseases, allowing for more targeted screening. Moreover, identifying common genetic factors can help in more accurate and multidimensional diagnosis and be effective in the detection of overlapping features between the two diseases. They can be effective in developing polygenic risk scores, which can help physicians in clinical decision‐making and predicting the likelihood of developing the disease. In addition, they may be applied in preventive interventions tailored to an individual's genetic profile or in the design of personalized treatment, as common genetic factors can indicate new drug targets or similar molecular pathways. Furthermore, these structures potentially could be incorporated into clinical genetic panels to examine the likelihood of developing the disease and help in clinical decisions.

In conclusion, this study identified the variant rs13218591 in the BTN3A1 gene and rs75782365 in BTN3A2 gene as two key genetic variants shared between schizophrenia and depression. Furthermore, two haplotypic structures, GTCG and GG, were characterized as components of a novel combined genetic framework predictive of the co‐occurrence of these disorders. The newly identified genetic architecture shows promise for potential inclusion in genetic screening panels designed to assess risk for depressive disorders, schizophrenia, or their comorbid occurrence. However, further validation through large‐scale and functional studies is necessary to confirm its predictive utility. Future research should focus on elucidating the biological mechanisms underlying these variants and haplotypic structures, as well as evaluating their effectiveness in clinical risk assessment and personalized treatment approaches.

## Author Contributions

**Morteza Gholami**: conceptualization, investigation, writing – original draft, writing – review and editing, methodology, validation, project administration, formal analysis, software, data curation, resources, supervision, visualization, funding acquisition.

## Funding

The author has nothing to report.

## Conflicts of Interest

The author declares no conflict of interest.

## Supporting information




**Supplementary Material**: brb371554‐sup‐0001‐SuppMat.xlsx

**Supplementary Material**: brb371554‐sup‐0002‐SuppMat.xlsx

**Supplementary Material**: brb371554‐sup‐0003‐SuppMat.docx

## Data Availability

The data that supports the findings of this study are available in the supplementary material of this article.
