# Supplementary material for: Identification of Shared Genetic Variants and Haplotypes Associated With Schizophrenia and Depression
Source: Brain Behav. 2026 Jun 14;16(6):e71554. doi: 10.1002/brb3.71554 (PMC13265835; doi:10.1002/brb3.71554)
Supplement: Supplementary file 3 — Supplementary Material: brb371554‐sup‐0003‐SuppMat.docx [file BRB3-16-e71554-s003.docx]

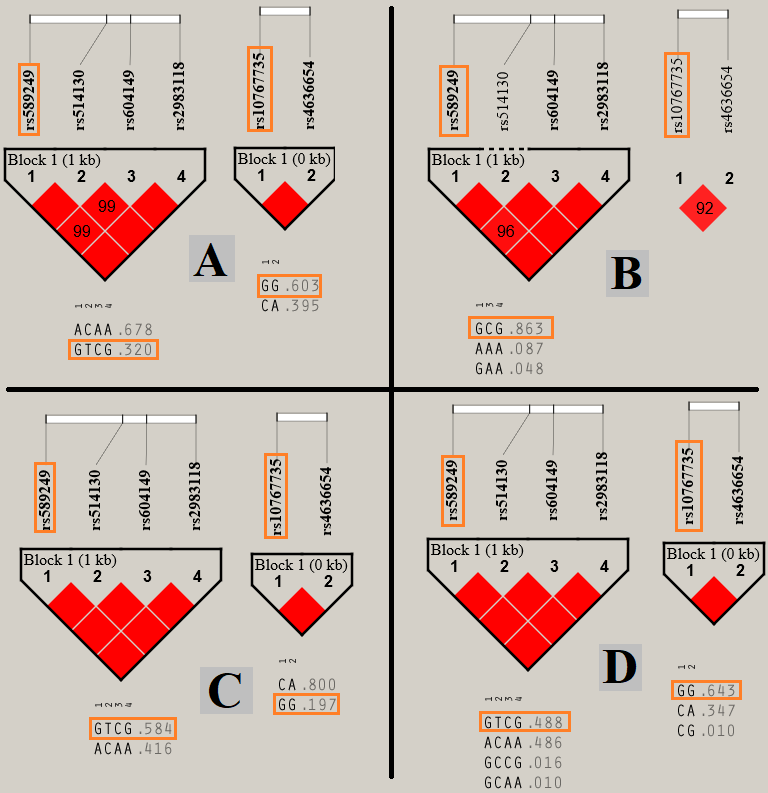


Supplementary Figure. LD plots and haplotypic structures common between schizophrenia and depressive disorder. A. European population B. African population C. Asian population D. American population
